# Adenomyosis: Difficult to Diagnose, and Difficult to Treat

**DOI:** 10.1155/DTE.7.89

**Published:** 2001

**Authors:** C. Wood

**Affiliations:** Endometriosis Care Centre of Australia Monash IVF Monash University Melbourne Victoria Australia

## Abstract

Drug therapy may be effective in controlling symptoms but the frequent coexistence of endometriosis and the lack of controlled studies make their efficacy difficult to quantify. Danazol IUD has been shown to reduce symptoms. Conservative surgery involving endomyometrial ablation, laparoscopic myometrial electrocoagulation or excision has proven to be effective in more than 50% of patients, although follow up has been restricted to three years. Arterial uterine artery embolization is a new technique which may be tried before considering hysterectomy. Hysterectomy may still be necessary in severe cases of adenomyosis.


*Diagnostic and Therapeutic Endoscopy*, Vol. 7, pp. 89–95
Reprints available directly from the publisher
Photocopying permitted by license only

© 2001 OPA (Overseas Publishers Association) N.V.
Published by license under
the Harwood Academic Publishers imprint,
part of Gordon and Breach Publishing,
a member of the Taylor & Francis Group.

*Short Communication*

### Adenomyosis: Difficult to Diagnose, and Difficult to Treat

C. WOOD*

*Endometriosis Care Centre of Australia, Monash IVF, Monash University, Melbourne, Victoria, Australia*

*(Received 16 June 2000; Revised 14 August 2000; In final form 4 September 2000)*

**Drug therapy may be effective in controlling symptoms but the frequent coexistence of endometriosis and the lack of controlled studies make their efficacy difficult to quantify. Danazol IUD has been shown to reduce symptoms. Conservative surgery involving endomyometrial ablation, laparoscopic myometrial electrocoagulation or excision has proven to be effective in more than 50% of patients, although follow up has been restricted to three years. Arterial uterine artery embolization is a new technique which may be tried before considering hysterectomy. Hysterectomy may still be necessary in severe cases of adenomyosis.**

*Keywords*: Adenomyosis, Treatment

### INTRODUCTION

The treatment of adenomyosis has been limited by the difficulty and delay associated with the diagnosis, often not until after hysterectomy. Magnetic resonance imaging, high resolution vaginal ultrasound and uterine biopsy has improved early detection of adenomyosis [1].

Diagnosis of the extent and localization of the disease is difficult, even with magnetic resonance imaging (MRI), colour doppler vaginal ultrasound and uterine biopsy techniques [2–9]. MRI is the most sensitive test for detection of adenomyosis but it is also the most expensive, limiting access to affluent health care systems [8]. Uncertainty in defining the site and more particularly the extent of adenomyosis make it difficult to determine the feasibility and accuracy of complete excision when conserving the uterus. This is one reason why hysterectomy has remained the most popular operation for adenomyosis.CA125 may be useful in diagnosis or determining the extent of the disease [10,11].

In subfertile women, under the age of 30 years, with dysmenorrhoea and menorrhagia, 28 of 56

* Tel.: +61 3 9415 7722. Fax: +61 3 9415 8461.

women having MRI and uterine histology have adenomyosis [12].

One of the difficulties assessing results of therapy is the lack of certainty of diagnosis, even with histology, because of false negatives, and uncertainty of the MRI criteria for diagnosis. The specificity of the diagnosis by MRI, has been determined by a junctional zone thicker than 5 mm. Thickness greater than 5 mm has been found in 40% of normal subjects having serial MRI measurements which also showed thickening up to 12 mm and focal myometrial bulging which may result from uterine contractions [13]. Myometrial changes in thickness, nodularity and asymmetry, and increased vascularization may be seen at MRI and sensitive vaginal ultrasound.

Drug therapy may be effective in controlling symptoms but the frequent coexistence of endometriosis and the lack of controlled studies make their efficacy difficult to quantify. Danazol IUD has been shown to reduce symptoms.

### SPECIFIC ENDOSCOPIC SURGICAL TECHNIQUES

The choice of a suitable surgical procedure depends upon the site and extent of disease, the age of the patient, the desire for future pregnancy, the patient's desire for certain cure or not, and the surgical skill of the gynaecologist.

### Endo-myometrial Ablation/Resection

Endo-myometrial resection is most suited to patients with disease limited to the endo-myometrial junction as menstrual symptoms may be reduced and the pathology may be removed. It may also be useful when adenomyosis is present in the outer myometrium as laparoscopic myometrial excision alone may not cure menstrual symptoms, either because excision may be incomplete or the menstrual symptoms are not caused by the outer myometrial adenomyosis. Desire for a future pregnancy contraindicates endo-myometrial resection.

#### *Technique*

The technique of endometrial ablation has been well described. If MRI or ultrasound shows the extent and site of endo-myometrial distortion the procedure can be modified to include 2–3 mm of myometrium in the affected areas. The whole of the endometrium should be removed as menorrhagia may be due to factors other than the adenomyosis. Deeper myometrial removal or ablation carries the risk of causing increased bleeding as significant arteries are situated about 5 mm deep to the myometrial surface. Histology of the excised myometrial fragments may help to confirm or refute the diagnosis.

When endo-myometrial resection has been performed as a single operative procedure, menstrual symptoms have been controlled in 55% of women for at least 2 years (Table I).

### Laparoscopic Myometrial Electrocoagulation

Electrocoagulation has the capability of shrinking adenomyosis by causing necrosis. The technique has been applied to localized or extensive disease

TABLE I Results of conservative surgery, 1991–97

| | Symptom free | | | Hospital stay | Complications |
|---|---|---|---|---|---|
| | *N* | 6 mths | 24 mths | (days) | |
| Endo-myometrial resection | 18 | 12 | 10 (55%) | 1 | 0 |
| Myometrial electrocoagulation | 11 | 7 | 6 (55%) | 1–2 | 0 |
| Myometrial excision | 25 | 20 | 16 (64%) | 1–3 | 3* |
| Subsequent hysterectomy | | | 10/54 (18.5%) | | |

* 3 patients had a temperature of > 37.5° for more than 2 days.

[1]. The adenomyosis can be detected by MRI, vaginal ultrasound, inspection of the uterus at laparoscopy, myometrial needling, or manual palpation during gasless laparoscopy to detect differences in consistency between normal and abnormal tissue. Electrocoagulation may be less accurate than surgical excision as electrical conduction in the abnormal tissue may be incomplete and this cannot be checked at the time of surgery. It may also reduce the strength of the myometrium by replacing abnormal myometrium with scar tissue. The width of the scar may be more extensive than after surgical excision when close apposition of normal myometrium is achieved.

Extensive myometrial electrocoagulation has been performed in two women aged 42 and 46 years with extensive adenomyosis on the anterior and posterior uterine walls; drug therapy had failed, excision was not feasible, and hysterectomy was not wanted. Two years later both are free of severe menstrual pain and bleeding. Diffuse multifocal electrocoagulation of the myometrium containing adenomyosis may be sufficient to control symptoms. The risk of uterine rupture following extensive electrocoagulation is demonstrated by the following experience. One patient had two laparoscopic procedures involving myometrial electrocoagulation, one of which was also associated with excision of an elevated adenomyotic area. The patient was aware of the risk of uterine rupture, she had not responded to GnRH analogue therapy, was not suitable for extensive myometrial excision and had refused hysterectomy as she wished to attempt conception even if this failed. A subsequent pregnancy resulted in uterine rupture at 12 weeks.

Electrocoagulation is best suited to women over 40 years of age, who do not wish to conceive, and who wish to avoid more extensive surgery such as excision or hysterectomy. Even if recurrence occurs the procedure may be repeated until the onset of the menopause when symptoms cease.

### *Technique*

Uterine manipulation with a Valtchev manipulator improves access to the diseased areas by facilitating antero-posterior and lateral movement of the uterus.

Vasoconstricting agents such as adrenaline and vasopressin are not used routinely as excessive bleeding has not been experienced and the blanching of the myometrium after vasoconstriction makes it difficult to determine the devascularizing effect of electrocoagulation or uterine vessel closure.

Closure of the ascending uterine artery may be performed if technically feasible, future pregnancy is not wanted, and the site of the adenomyosis is in the upper uterine body. Bipolar forceps, clips or suture ligation may be used to close the uterine vessels.

Electrocoagulation of the adenomyosis may be carried out with unipolar or bipolar needles, using 50 watts coagulation current. Bipolar needles have a theoretical advantage of concentrating current between the two needles, but their effectiveness is diminished by the tendency of the two needles to move close together as they penetrate the myometrium. Additionally, the area of coagulation may spread outwards from each needle, simulating the effect of monopolar electrocoagulation.

The extent of coagulation can be controlled by reducing the current strength and changing the time the needle(s) are held in position. In order to reduce the possibility of severe surface necrosis and carbonization, either of which may encourage future adhesion formation, the insulated part of the needle is buried a few millimetres below the uterine surface before electrocoagulation is commenced. The insulation on the bipolar needle can be extended so that the active part of the electrode is shortened in order to avoid surface coagulation and necrosis. Needle punctures are made at 1–2 cm intervals, depending on the spread of the coagulative effect. The depth of needle puncture may vary, depending on the thickness of the adenomyotic myometrium determined preoperatively by ultrasound or MRI. If hysteroscopic endo-myometrial ablation has also been carried out, the depth of laparoscopic needle electrocoagulation may be reduced.

Hysteroscopic endo-myometrial ablation may be performed in association with myometrial electrocoagulation as menorrhagia and dysmenorrhoea may not be related to the presence of outer myometrial adenomyosis.

Sterilization should be offered to all women having myometrial electrocoagulation because of the possible future risk of uterine rupture in pregnancy.

Bleeding is rare during electrocoagulation and can be controlled by using a vasopressor or myotonic drugs such as adrenaline, oxytoxin or vasopressin, or by bipolar electrocoagulation or suture ligation.

Patients are usually in hospital for 8–24 hours. No complications have been observed including post-operative infection, bleeding or subsequent adhesion formation.

The result of the surgery may be assessed by symptom relief and MRI or vaginal ultrasound. Loss of features of adenomyosis including reduction of myometrial thickness, reduced vascularity and normal myometrial appearance have all been observed. Symptom relief may occur and persist for several years in the presence of reduced ultrasound evidence of adenomyosis.

### Myometrial Excision

Adenomyosis may be excised if it does not involve the major portion of the uterus, and its extent can be defined as previously described. The technique is also suitable for adenomyomas where the margins of the pathology are more easily defined. It may be useful in women wishing to become pregnant, providing sufficient myometrium remains to allow uterine expansion and term pregnancy and the scar formed after excision is not wide or shallow. MRI or colour doppler ultrasound after surgery should be used to check both for cure, the width and depth of scar, and the possible association of residual adenomyosis close to the scar, before attempts at conception are advised.

### *Technique*

Preoperative GnRH analogues or Danazol may reduce uterine vascularity, correct anaemia if the patient has severe menorrhagia, and reduce operative bleeding which facilitates surgery by laparoscopy rather than laparotomy. Vasoconstrictor drugs may also reduce bleeding at the time of surgery.

Prior to myometrial excision, as with electrosurgical coagulation, the uterine blood supply may be reduced by suture or clip ligation or bipolar diathermy of the ascending uterine vessels in women not concerned with fertility. Apart from reducing bleeding during surgery the reduction in blood flow may reduce future growth or development of adenomyosis.

Two associated surgical procedures may be offered, sterilization to prevent conception and hysteroscopic endomyometrial ablation if menorrhagia is present, and fertility is not required.

Minilaparotomy may be required to facilitate myometrial excision. Laparotomy instruments can gain entry to the abdomen through a 2–4 cm incision which may be sufficient to remove and repair areas of myometrium up to $6 \times 8$ cm.

A Valtchev uterine manipulator is used to position the adenomyotic areas as close as possible to a laparoscopic or minilaparotomy incision. Sometimes a myoma screw may stabilize the diseased area and aid excision. A diathermy spoon using 100 watts monopolar current, or scalpel, are suitable for excision. The spoon has the advantage of cutting effectively with the sharp end close to the tissue, and coagulating vessels when the convex curve of the spoon compresses the vessel. When the tissue is very firm the scalpel may be preferable, providing more effective and rapid excision. The margin of the adenomyosis may be determined by change in appearance, vascularity or consistency; finger palpation may be an advantage.

Closure of incisions longer than 5–6 cm may require laparotomy instruments as excision of a significant volume of myometrium increases the tension at the myometrial edges which may have to be stretched to close the defect. If the uterine wound is brought into a minilaparotomy incision, the defect can be closed more easily and quickly. Absorbable sutures (No. 1) are used in one or more layers. If there is a large defect a single layer through and through suture may best approximate the wound, acting as a tension suture, and because of the increased thickness of the whole myometrium, it is less likely to tear as tension is increased to attain closure.

Anti-adhesives such as Interceed® and Goretex® membrane may be used. The frequency of adhesions after excision of adenomyosis has not been reported. Interceed may be used if perfect haemostasis is obtained. Application of Surgicel® prior to Interceed may improve haemostasis and allow the use of Interceed. If bleeding persists Goretex can be stapled over the wound. This need not be removed unless pregnancy is planned. Uterine enlargement may displace the membrane from the uterus which may attach to other organs.

Hysterectomy may still be necessary in severe cases of adenomyosis. Severity of adenomyosis is related to late diagnosis. Early diagnosis may improve treatment and investigations are indicated in women with menstrual pain or menorrhagia not responding to drug therapy.

### Results of Conservative Surgery

Conservative surgery in a personal series of 62 women with adenomyosis and diagnosed by vaginal ultrasound with doppler assessment, percutaneous uterine needle biopsy, and histology of excised endo-myometrial or needle myometrial biopsy at the time of hysteroscopy or laparoscopy, resulted in 63% of women being symptom free 2 years later and 12% requiring hysterectomy during the same time period because of persistence or recurrence of severe symptoms. Each of the techniques had a success rate greater than 50%. Nine of 16 women attempting pregnancy conceived, 4 of 7 after myometrial electrocoagulation and 5 of 9 after myometrial excision. One woman who had two electrocoagulation treatments, including one associated myometrial excision, subsequently ruptured her uterus in the twelfth week of pregnancy.

MRI and uterine biopsy were used to diagnose nodular adenomyosis by Phillips *et al.* [14]. Preoperative GnRH analogue, endomyometrial resection and bipolar coagulation were used in 14 women. One year after treatment menorrhagia was cured in 12 and dysmenorrhoea in 8. Two proceeded to hysterectomy. The advantage of preoperative use of GnRH analogue was shown by a 50.8% mean reduction of uterine volume after leuprolide acetate treatment for 3 months.

### GnRH Analogues

A GnRH analogue, leuprolide acetate has been used to produce a constant hypoestrogenic state in a woman with histologically proven adenomyosis [14]. Dysmenorrhoea and desire for conception were the two complaints. This produced amenorrhoea, control of pain and uterine shrinkage, and conception resulted.

A pure antiestrogen may offer some advantage in the treatment of adenomyosis and trials are planned to assess its usefulness in the human.

### Topical Danazol/Progestogen Therapy

Adenomyosis has been treated by 200 mg of danazol contained in an intrauterine device (DIUD) [15]. Blood danazol levels are undetectable, ovulation was not inhibited, and side effects did not occur. The DIUD was effective in 9 of 10 cases in reducing uterine size and dysmenorrhoea and pregnancy occurred in 3 cases after removal of the DIUD. Another study of the DIUD containing 300 µg of danazol produced similar results over 6–12 months [10]. Symptoms improved in more than 70% of patients especially for dysmenorrhoea, the DIUD was shown to be active after

12 months use, mean CA125 levels decreased from 295 U/ml to 115 U/ml, and mean uterine volume decreased from 369 $cm^3$ to 264 $cm^3$. Using the DIUD in Australian women 3 of 4 expelled the DIUD and a larger IUD would be required to test its efficacy.

The levonorgestrel intrauterine device (LNIUD) has proven to be effective not only as a contraceptive but also in the control of menorrhagia. Its action is to produce an atrophic endometrium. It may be useful in the control of menorrhagia in the presence of adenomyosis, and possibly the reduction of dysmenorrhoea. The antioestrogenic effect may reduce the growth of the adenomyotic tissue. A study of 25 women with menorrhagia associated with adenomyosis diagnosed by vaginal ultrasound has shown that 23 had relief of menorrhagia persisting for 1 year after use of the LNIUD [16]. Reduction of menstrual pain was less frequent. Spotting in the first 3 months was the most common side effect, one patient asking to have the device removed because of this. Six patients reported headaches, three breast tenderness, six greasy hair, seborrhoea or acne, and seven weight gain. Spotting was well tolerated.

### Arterial Embolization

Arterial uterine artery embolization has the potential to reduce blood flow to part or the whole of the uterus. Two patients with diffuse adenomyosis have been treated by embolization with cure or control of menstrual pain and menorrhagia; follow up is only short term. Angiography and doppler ultrasound demonstrates more than 50% reduction of uterine blood flow. The technique will be tried on all patients who would otherwise require hysterectomy. In both patients the posterior myometrial thickness was reduced by 45 and 58%, small myometrial cystic spaces were present in one and myometrial scarring increased in the other.

The major advantage of arterial embolization is that the angiogram localizes the site of adenomyosis by indicating increased vascularization often associated with adenomyotic areas, which can then be targeted by precise embolization of the arteries supplying the involved areas.

It may not be suitable for patients wishing to conceive, as large areas of scarred myometrium may weaken the uterus and result in uterine rupture in pregnancy.

The limitation of uterine artery embolization is the possibility of early regrowth of the adenomyosis and the limited availability of the technique. Its increasing use in treating certain types of fibroids may encourage exploration of the techniques' effectiveness or not in adenomyosis. Complications are unproven. The cure rate for menorrhagia in women with fibroids is more than 50% [17,18]. Pain may occur for up to 1–2 months which is similar to that after laparoscopic excision of adenomyosis. The technique has to be done by skilled radiologists e.g. those already treating brain aneurysms. Two cases in a review of all 2000 cases performed in the USA resulted in ovarian failure in three patients.

### *References*

[1] Wood, C. Surgical and Medical Treatment of Adenomyosis. *Hum. Reprod. Update* 1998; **4**(4): 323–336.

[2] Wood, C., Maher, P. and Hill, D. Biopsy Diagnosis and Conservative Surgical Treatment of Adenomyosis. *J. Am. Assoc. Gynecol. Laparosc.* 1994; **1**(4): 313–316.

[3] Brosens, J.J., de Souza, N.M., Barker, F.G. *et al.* Endovaginal ultrasonography in the diagnosis of adenomyosis uteri: identifying the predictive characteristics. *Br. J. Obstet. Gynaecol.* 1995; **102**(6): 471–474.

[4] Fedele, L., Bianchi, S., Dorta, M. *et al.* Transvaginal ultrasonography in the diagnosis of diffuse adenomyosis. *Fertil. Steril.* 1992; **58**(1): 94–97.

[5] McCausland, A.M. Hysteroscopic myometrial biopsy: its use in diagnosing adenomyosis and its clinical application. *Am. J. Obstet. Gynecol.* 1992; **166**: 1619–1626.

[6] Popp, L.W., Schwiedessen, J.P. and Gaetje, R. Myometrial biopsy in the diagnosis of adenomyosis uteri. *Am. J. Obstet. Gynecol.* 1993; **169**(3): 546–549.

[7] Kang, S., Turner, D.A., Foster, G.S. *et al.* Adenomyosis: specificity of 5 mm as the maximum normal uterine junctional zone thickness in MR images. *Am. J. Roentgenol.* 1996; **166**(5): 1145–1150.

[8] Reinhold, C., McCarthy, S., Bret, P.M. *et al.* Diffuse adenomyosis: comparison of endovaginal US and MR imaging with histopathologic correlation. *Radiology* 1996; **199**(1): 151–158.

[9] Brosens, J.J. and Barker, F.G. The role of myometrial needle biopsies in the diagnosis of adenomyosis. *Fertil. Steril.* June 1995; **63**(6): 1347–1349.

[10] Tanaoka, Y., Slo, R., Yamamoto, Y. *et al.* Investigation of treatment of adenomyosis by intrauterine device that contains danazol. Fifth World Congress on Endometriosis. Abstracts 21–24 October, Yokohama, Japan 0-156, 1996, p. 80.

[11] Koninckx, P.R., Riittinen, L., Sepälä, M. and Cornillie, F.J. CA-125 and placental protein 14 concentration in plasma and peritoneal fluid of women with deeply infiltrating pelvic endometriosis. *Fertil. Steril.* 1992; **57**: 523–530.

[12] Brosens, J.J., de Souza, R.N. and Barker, F.G. Uterine junctional zone: function and disease. *Lancet*, August 26, 1995; **346**: 558–560.

[13] Kang, S., Turner, D.A., Foster, G.S. *et al.* Adenomyosis: specificity of 5 mm as the maximum normal uterine junctional zone thickness in MR images. *Am. J. Roentgenol.* 1996; **166**(5): 1145–1150.

[14] Phillips, D.R., Nathanson, H.G., Milim, S.J. *et al.* Laparoscopic bipolar coagulation for the conservative treatment of adenomyomata. *J. Am. Assoc. Gynecol. Laparosc.* 1996; **4**(1): 19–24.

[15] Igarashi, M., Iizuka, M., Ahe, Y., *et al.* A new therapy for uterine adenomyosis. Fifth World Congress on Endometrisois. Abstracts 21–24 October, Yokohama, Japan 0-155, 1996.

[16] Fedele, L., Bianchi, S., Raffaeli, R. *et al.* Treatment of adenomyosis – associated menorrhagia with a levonorgestrel-releasing intrauterine device. *Fertil. Steril.* 1997; **68**(3): 426–429.

[17] Hutchins, F.L. Jr., and Worthington-Kirsh, R.L. Initial experience with uterine artery embolization for the management of symptomatic uterine fibroids. *J. Am. Assoc. Gynecol. Laparosc.* 1997; **4**: 27.

[18] Vedanthen, S., Goodwin, S.C., McLucas, B. and Forno, A.E. Uterine artery embolization for uterine fibroids. *J. Am. Assoc. Gynecol. Laparosc.* 1997; **4**: 39.

